# Bronchus Cardiacus Accessorius Dexter

**DOI:** 10.1155/DTE.5.211

**Published:** 1999

**Authors:** P. Barzó, B. Nagy

**Affiliations:** 1 Third Department of Pulmonology St. Ferenc Hospital Miskolc H-3501 Hungary; 2 University Medical School of Debrecen Pediatric Clinic Debrecen H-4012 Hungary

## Abstract

The diagnosis of bronchus cardiacus accessorius dexter (BCAD) has occurred in 25 cases during the bronchoscopic investigations of 30,000 adult patients of the authors. In most of the cases, this bronchial anomaly has been revealed as an accessory phenomenon, nevertheless, in one of the patients, it was the source of a considerable hemorrhage. In another case reported here in detail, it occurred together with multiple developmental anomalies, such as tracheobronchomegaly, mitral valve prolapse, pectus excavatum, hypoplasy of sinus frontalis on the right side, inguinal hernia on the left side and hyperlipidemia type IV. Family analysis did not confirm the presence of any chromosomal disorders or accumulation of similar developmental anomalies. The forms and frequency of associations of the anomalies are surveyed on the basis of literary data. The recognition of BCAD is of diagnostic importance, since it may explain the persistence of some bronchopulmonary symptoms; furthermore, the exploration of the associated abnormal vascular branches may be very useful in case of an eventual thoracic surgical intervention.


*Diagnostic and Therapeutic Endoscopy*, Vol. 5, pp. 211–217
Reprints available directly from the publisher
Photocopying permitted by license only

© 1999 OPA (Overseas Publishers Association) N.V.
Published by license under
the Harwood Academic Publishers imprint,
part of The Gordon and Breach Publishing Group.
Printed in Malaysia.

### Bronchus Cardiacus Accessorius Dexter

P. BARZÓ[a,*] and B. NAGY[b]

[a] *Third Department of Pulmonology, St. Ferenc Hospital, H-3501 Miskolc, Hungary;*
[b] *University Medical School of Debrecen, Pediatric Clinic, H-4012 Debrecen, Hungary*

*(Received 12 February 1998; Revised 10 July 1998)*

**The diagnosis of bronchus cardiacus accessorius dexter (BCAD) has occurred in 25 cases during the bronchoscopic investigations of 30,000 adult patients of the authors. In most of the cases, this bronchial anomaly has been revealed as an accessory phenomenon, nevertheless, in one of the patients, it was the source of a considerable hemorrhage. In another case reported here in detail, it occurred together with multiple developmental anomalies, such as tracheobronchomegaly, mitral valve prolapse, pectus excavatum, hypoplasy of sinus frontalis on the right side, inguinal hernia on the left side and hyperlipidemia type IV. Family analysis did not confirm the presence of any chromosomal disorders or accumulation of similar developmental anomalies. The forms and frequency of associations of the anomalies are surveyed on the basis of literary data. The recognition of BCAD is of diagnostic importance, since it may explain the persistence of some bronchopulmonary symptoms; furthermore, the exploration of the associated abnormal vascular branches may be very useful in case of an eventual thoracic surgical intervention.**

*Keywords:* Bronchus cardiacus accessorius dexter, Tracheobronchomegaly, Pectus excavatum, Mitral valve prolapse, Sinus frontalis hypoplasy

### INTRODUCTION

The bronchus cardiacus accessorius dexter (BCAD) is a rare bronchial anomaly. It occurs in the form of an opening of the size of a segmental bronchus, localized most frequently opposite to the origin of the right upper lobe bronchus, or somewhat more distally to it. Exceptionally, it may originate also from the lower part of the truncus intermedius. On the bronchograms it appears as a blind sack of medio-downward direction, bearing sometimes also a small, respiring pulmonary lobule [1–4]. Its presence can be proven, if we can identify the two segmental branches of the middle lobe bronchus, and all segmental branches of the lower lobe [5]. The literature is relatively neglecting this bronchial anomaly, although its recognition might explain a number of persisting bronchopulmonary symptoms.

* Corresponding author.

### PATIENTS AND A CASE REPORT

Our material derives from 30,000 adult bronchoscopic investigations: BCAD was detected in 25 cases (6 women and 19 men). Bronchoscopy was indicated by various acute and chronic diseases of the respiratory tract. In 6 out of 25 patients, BCAD originated from the lower part of the truncus intermedius. Among the associated pathologies, one can list 11 cases of obstructive bronchitis, 4 cases of pneumonia, and 2 cases each of epidermoid carcinoma, bronchial asthma, pulmonary tuberculosis and tuberculotic bronchoadenitis. A small pulmonary lobule was present on the BCAD only in 6 cases. In a 51-year-old women, a rudimentary BCAD of cranial localization caused a severe hemoptysis. Our investigations revealed that the bleeding was caused by the vaso-ulcerative, destructive effect of a tuberculotic, in part calcified lymphnode of adjacent localization.

The case of our patient (27-year-old man) deserves a detailed description, because in his case, BCAD was accompanied with multiple congenital anomalies. This patient was a photographer, who was sent to our department because of some chronic symptoms, like slight coughing, morning expectoration, and frequent nasal discharge. He was a slow growing, sickly child, who could catch up with the age-matched boys in his development only at about 12 years of age. In his anamnesis, we recorded tonsillectomy because of chronic tonsillitis, left sided inguinal herniotomy, and a surgical correction of pectus excavatum which had been strongly expressed since the infancy (Fig. 1).

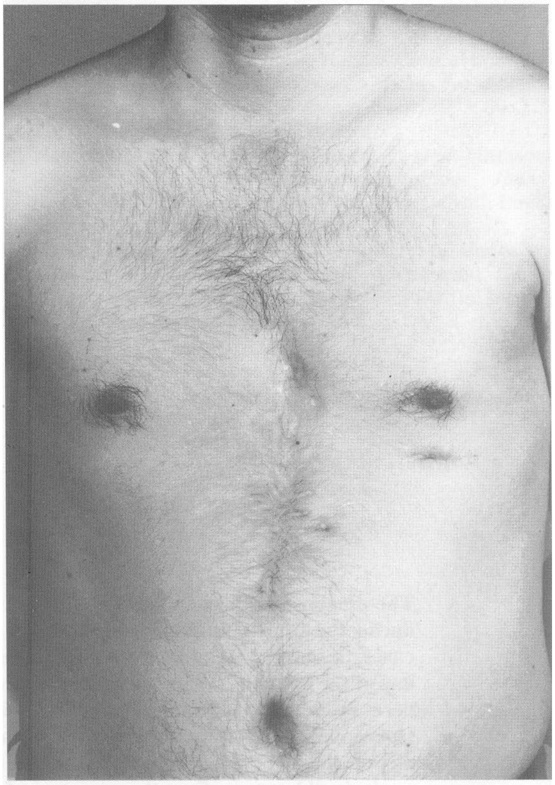

FIGURE 1 Corrected pectus excavatum.

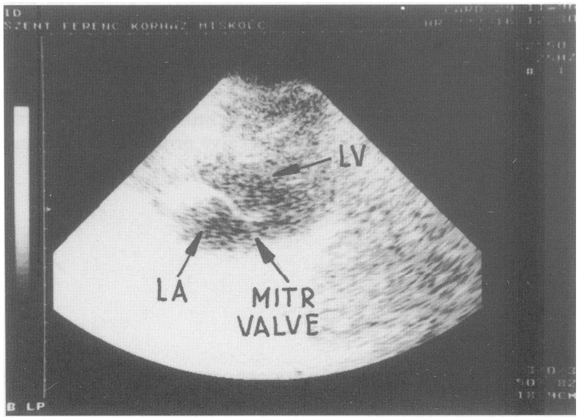

FIGURE 2 The telesystolic prolapse of anterior part of the mitral valve.

From the physical findings, one can mention the saddle-nose, the scars of the above mentioned surgeries, and a weak systolic murmur above all cardiac valves. The latter proved to be of ejection type by means of phonocardiographic (PCG) examination. Electrocardiograms (ECG) displayed ventricular extrasystoles (ES), and echocardiography revealed the mitral valve prolapse (Fig. 2). Chest X-ray pictures did not reveal any pathological alteration. Bronchoscopy discovered the presence of a typical BCAD of cranial localization, together with the signs of a chronic bronchitis (Fig. 3). According to the bronchographic findings, a rudimentary lobule was also in connection with the accessory cardiac bronchus. The trachea and the

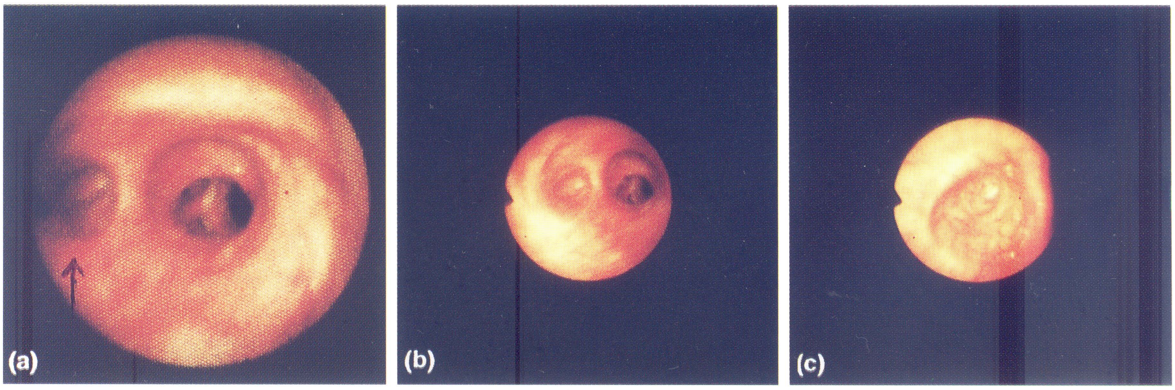

FIGURE 3 The bronchoscopic image of a BCAD.

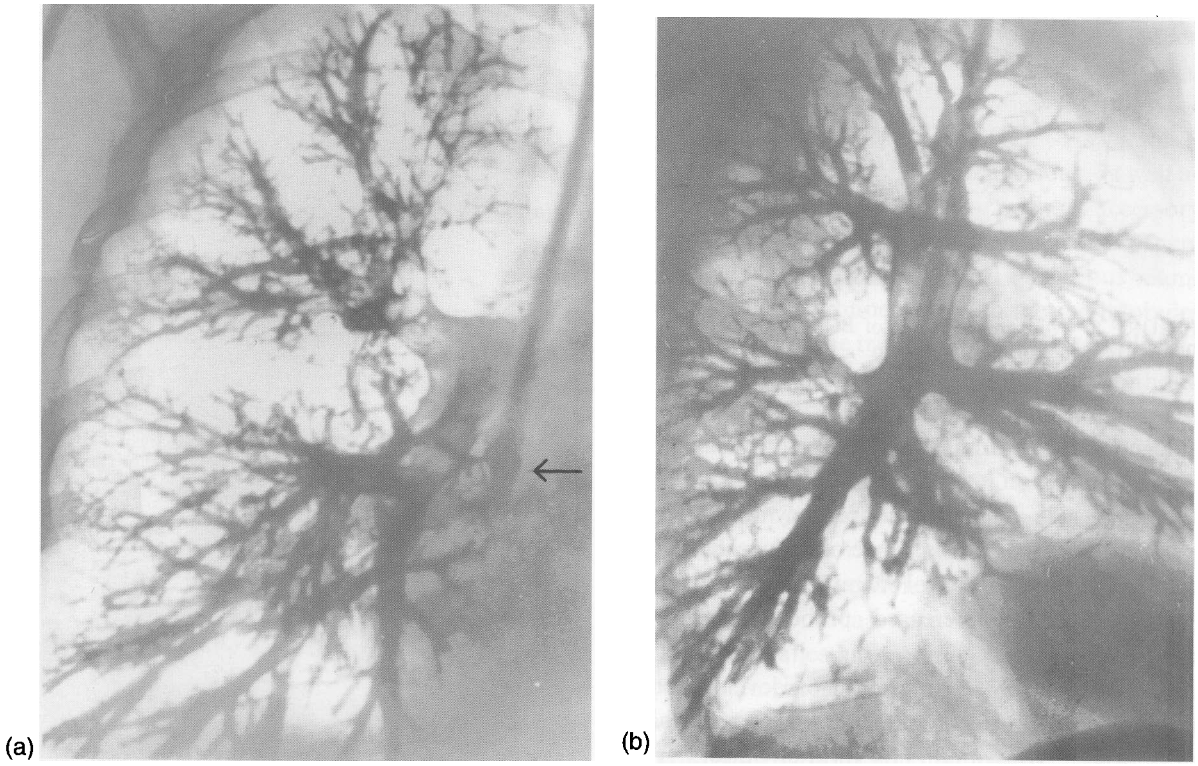

FIGURE 4 A: The site of BCAD indicated by the catheter on the right posteroanterior bronchogram. B: Bronchogram of lateral direction. Both pictures demonstrate a moderate widening of the lumen of the larger bronchial branches.

main bronchi were considerably wider than the normal, and the pattern of the wall cartilages was well expressed, however, no substantial destruction, diverticuli or dyskinesia could be observed (Figs. 4–6). Scintigraphic examination of the lung indicated a somewhat decreased perfusion in the right apex pulmonis, although the analysis of the respiratory functions could not prove any pathological alteration. X-rays of the nasal sinuses displayed a rudimentary sinus frontalis on the right

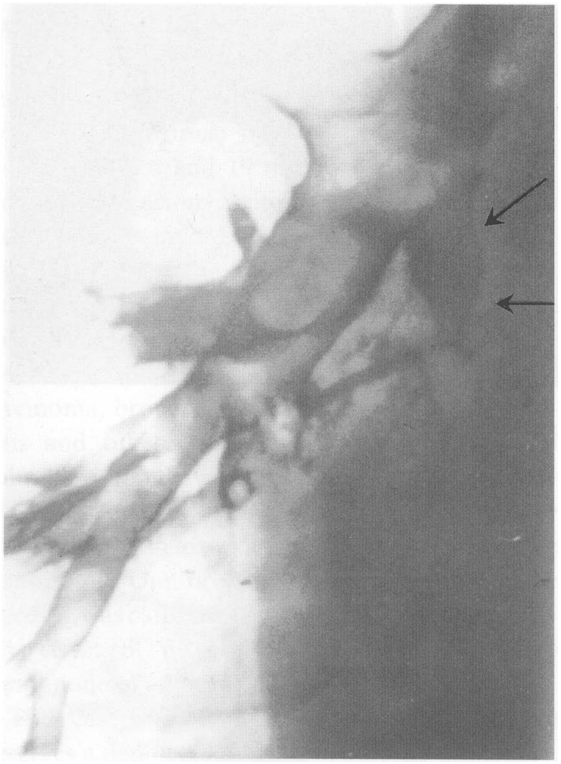

FIGURE 5 A magnified image of BCAD together with the filiform branches of the lobule of it.

side, containing radially thickened mucosa in chronic inflammation, as well as a septum deviating to the right side (Fig. 7). Laboratory data of the patient indicated the existence of a hyperlipidemia type IV; serum cholesterol 8.0 mmole/L, triglycerids 9.2 mmole/L, an increased prebeta lipoprotein and VLDL.

Because of the accumulation of developmental anomalies, the family members of the patient have also been investigated. His mother suffers from psoriasis, while his father and his father's brother are healthy. His sister and her children are also healthy. His brother bears a combined developmental anomaly of the large blood vessels, detected at the age of 5 years: the truncus brachiocephalicus and the carotis communis dexter originate with a joint truncus from the aorta, nevertheless, this does not cause any symptoms or complaints. The grandparents were treated because of tuberculosis,

(a)

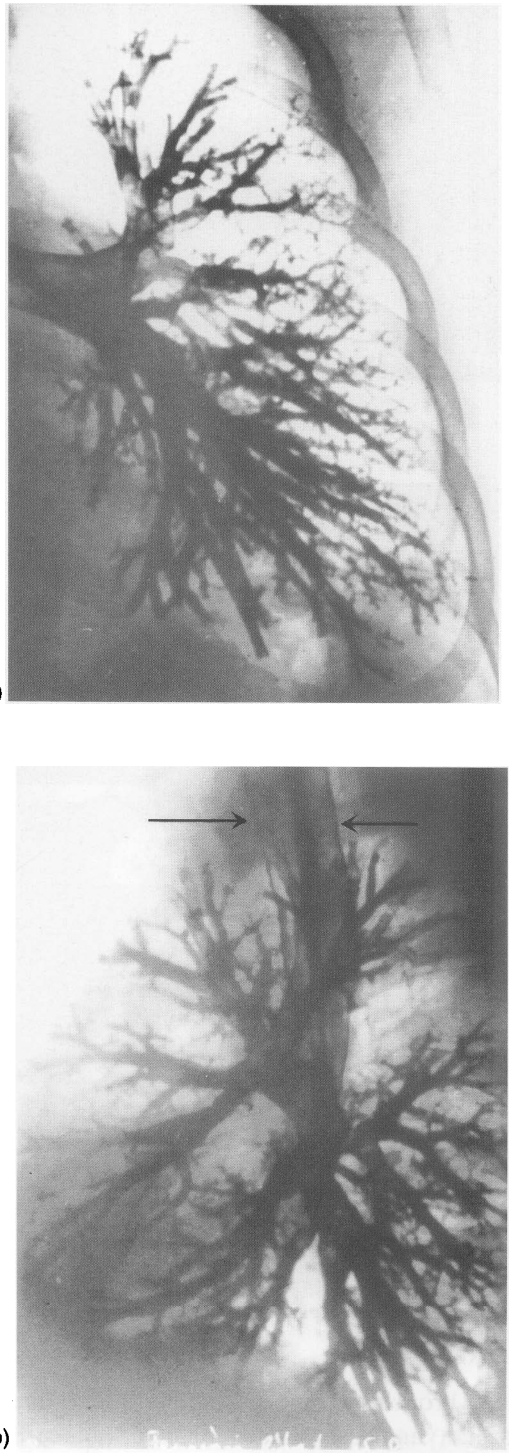

(b)

FIGURE 6 A: Left posteroanterior bronchogram. B: Bronchogram of lateral direction. Both of them show the larger sizes of trachea and the left main bronchus.

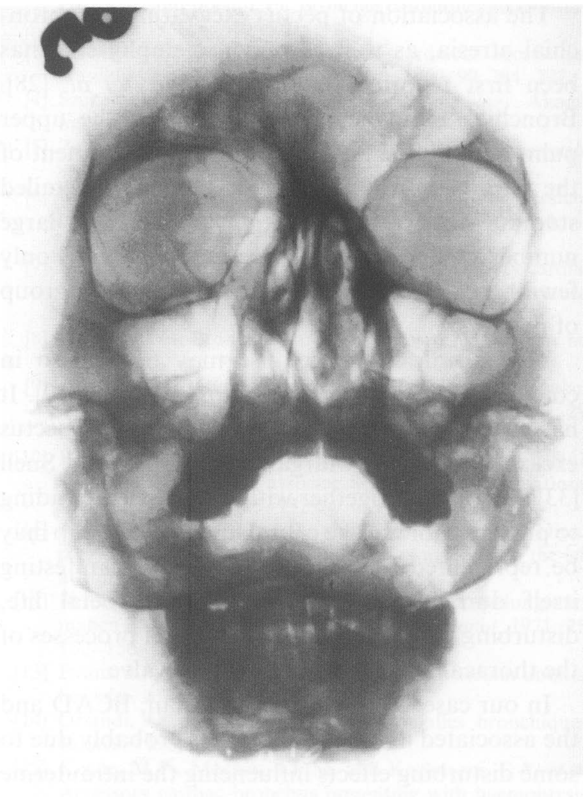

FIGURE 7 The hypoplasy of the right sinus frontalis.

bronchial asthma and heart attack. The father's side grandparents had two daughters and one son who died during the early babyhood; the same happened to one son of the father's side aunt. The karyotypes of the patient, his mother and his sister proved to be normal.

### DISCUSSION

The BCAD as a bronchial anomaly has been described first by Brock; Lemoine and Gagnon, as well as by Kato [cit. 2,6,7], however, the name of it was given by Huzly and Boehm [2]. Its occurrence was established between 0.1% and 0.46% by various authors [2,4–9], and this prevalence was observed also in our material.

The joint occurrence of BCAD and other bronchial anomalies has been known in the literature: it was observed together with right tracheal bronchus in 2 cases [7,10], with branching anomalies of the left bronchial system in 2 patients [2,6] with cardiovascular developmental anomalies in 1 case [11] and at last with arteriovenous shunt in 1 patient [5]. Huzly described it in 1960 on the left side in 1 patient examined because of situs inversus. The occurrence of 2 BCAD on the very same side has been observed only in 1 case [7].

The inheritance of BCAD is not clear. Its occurrence has been described also in both twins [8,9]. The mechanism of its formation is studied nowadays following the embryonic development of the lungs [12,13]. Namely, during 4–6th weeks of the intrauterine life, a primordial bronchus called lobulus infracardiacus grows out from the right bronchial anlage of the embryo, which disappears at later stages of development [12]. The lobulus intermedialis, the lobulus infracardiacus, as well as the bronchus cardiacus superior with its accessory lobule represent various forms of the same organ. Similar situations were observed in various mammalian species in connection with the small lobules originating from the truncus intermedius. Bolla and Zanotelli [10] in agreement with other authors, consider human BCAD as an involutionary rest of lobus azygos or lobus accessorius [6].

According to Zebrak [5], BCAD is not an isolated anomaly, but it is accompanied by other developmental disorders, or by acquired diseases, such as pulmonary cysts, facial fissures and bronchiectasy. Although in our case presented here in detail, various anomalies have occurred together, and the above statement has been confirmed neither by our other cases, nor by literary data [14]. One can assume that BCAD is self-standing in the genotype, and in cases of its association with other developmental anomalies, the additional malformations are due to some damage of the embryo during the 5th week [7,10].

Several authors called attention to the importance of differentiating between BCAD and the fistula-opening of tuberculotic bronchoadenitis [6,7]. The size of the opening, the frequently circular, annulus cartilaginous-like structure and the branching shape of the bronchogram may be of help in this. The BCAD may rarely give way to a bronchial perforation of tuberculotic lymphnode. In such cases one can see bleeding from the BCAD, as it occurred in one of our cases. Béguery *et al.* [6] and Bolla and Zonatelli [10] also described the bronchial irruption, however, apart from our observation, the origin of bleeding was proven endoscopically only by Keane *et al.* [15].

The diagnosis of tracheobronchomegaly (TBM) is based on bronchoscopy and bronchography, and more recently also on chest CT [16,17]. Its presence in adults can be established, if the antero-posterior diameter exceeds 25 mm, and the transversal diameter is larger than 30 mm, and the right and left main bronchi are wider than 20 mm [18,19]. In our patient, these parameters were 27, 32 and 23 mm, respectively, i.e., can be considered as pathognostic ones. Himalstein [18] classified this pathology in 3 subtypes: (i) a slight, symmetric widening of trachea and the main bronchi; (ii) expressed widening, with peculiar, excentric forms, and the presence of diverticula, and (iii) diverticula are present also on the distal bronchi.

Our patient can be classified in the first subtype which is a relatively benign form with slow pathological course [20], supported also by our experience of a 12-year follow-up.

The causes of TBM are unknown. The atrophy of the longitudinal elastic fibers of trachea is mainly congenital, however, the genetic background of this pathology is not clear [20,21]. It is present in numerous syndromes involving the weakness of the connective tissue, e.g., the Ehlers–Danlos, the cutis laxa, the Kenny–Caffey [22], the Klinefelter syndromes, immunodeficient diseases (ataxia teleangiectasica, agammaglobulinemia of Bruton) [17,23]. It may occur together with duplication of trachea, doubling of carina, tracheal trifurcation and bronchial developmental anomalies [21,24], and in all these pathologies it indicates severe connective tissue defects [16,21]. Its acquired form is very rare [25,26]. Trachea dilatation with pulmonary fibrosis [27] has been observed in singers [21].

The association of pectus excavatum and bronchial atresia, as well as regional emphysema has been first reported by van Klaveren *et al.* [28]. Bronchial anomalies were detected in the upper pulmonary lobes. A more precise establishment of the interrelationships will require further detailed studies, since compared to the relatively large number of children with pectus excavatum, only few bronchoscopic data are available in this group of patients.

The mitral valve prolapse may occur also in congenital or acquired respiratory diseases [29]. It has been described frequently together with pectus excavatum [30,31]. Margaliot *et al.* [32] and Snell [33] described it together with lung diseases leading to pneumothorax. The causal interrelationship may be represented by a damaging factor manifesting itself during the 35–42nd days of foetal life, disturbing together the developmental processes of the thoracal skeleton and the mitral valve.

In our case described here in detail, BCAD and the associated anomalies are most probably due to some disturbing effects influencing the intrauterine development of the connective tissue. The publication of these data is justified first by the fact that our experience was collected on one of the largest number of BCAD cases in the literature, and second by the importance of the possible associations of BCAD with some other pathologies. Its recognition and differential diagnosis is of great importance in the everyday practice, and may be of help in the realization of eventual thoracic surgical interventions.

### *Acknowledgement*

Authors express their thanks to the Genetic Laboratory of the Pediatric Clinic of the University Medical School of Debrecen, for their help in the genetic examinations.

### *References*

[1] Bellini, F., Garavaglia, C. and Romagnoli, M. Su due casi di bronchoinfracardiaco superiore. *Minerva Medica* 1962; **53**: 651–655.

[2] Huzly, A. and Boehm, Fr. Bronches cardiaques accessories. *Bronches* 1956; **6**: 540–550.
[3] Scheffler, H. Bronchus et lobulus cardiacus accessorius cranialis dexter. *Fortschr. Röntgenstr.* 1963; **99**: 294–297.
[4] Székely, E. and Farkas, E. *Pediatric Bronchology*. Akadémiai Kiadó. Budapest, 1978.
[5] Zebrak, J. Bronchus und Lobulus cardiacus accessorius cranialis. *Z. Erkr. Atm.* 1973; **138**: 373–378.
[6] Béguery, P., Denies, J.L. and de Voogd, A. La bronche cardiaque accessoire. A propos d'un cas. Revue de la littérature. *J. Radiol.* 1980; **61**: 69–73.
[7] Mangiulea, V.G. and Stinghe, R.V. The accessory cardiac bronchus. Bronchologic aspect and review of the literature. *Chest* 1968; **54**: 35–38.
[8] Gubbawy, H. Bronchus cardiacus accessorius superior bei zwillingen. *Prax. Pneumol.* 1974; **28**: 487–490.
[9] Gubbawy, H. and Hofmann, A. Verzweigungsanomalien des Tracheobronchialbaumes. *Prax. Pneumol.* 1975; **29**: 288–296.
[10] Bolla, A. and Zanotelli, F. Considérations sur le diverticule bronchique simple et associeé a d'autres malformations. *Bronches* 1967; **17**: 125–132.
[11] Abe, T., Kuribayashi, R. and Sato, M. *et al.* Direct communication of the right pulmonary artery with the left atrium. *J. Thor. Cardiovasc.* 1972; **64**: 3844.
[12] Bereti, I. and Hordós, A. Seltene Bronchusteilungsanomalien als atavistische Zeichen. *Prax. Pneumol.* 1971; **25**: 152–156.
[13] Evans, J.A. Abberrant bronchi and cardiovascular anomalies. *Am. J. Med. Genet.* 1985; **21**: 21–34.
[14] Orlandi, O. and Ferrero, P.G. Anomalies bronchiques. *Bronches* 1962; **12**: 439–465.
[15] Keane, M.P., Meaney, J.F.M. and Kazerooni, E.A. *et al.* Accessory cardiac bronchus presenting with haemoptysis. *Thorax* 1997; **52**: 490–491.
[16] Goh, R.H., Dobranowsky, J. and Kahana, L. *et al.* Dynamic computed tomography evaluation of tracheobronchomegaly. *Can. Assoc. Radiol.* 1995; **46**: 212–215.
[17] van Schoor, J., Joos, G. and Pauwels, R. Tracheobronchomegaly – the Mounier–Kuhn syndrome: report of two cases and review of the literature. *Eur. Respir. J.* 1991; **4**: 1303–1306.
[18] Himalstein, M.R. and Gallagher, J.C. Tracheobronchomegaly. *Ann. Otol.* 1973; **82**: 223–227.
[19] Onorato, D.J. and Harrison, P. Tracheobronchomegaly: a rare view from below. *J. Bronchol.* 1996; **3**: 280–282.
[20] Johnston, R.F. and Green, R.A. Tracheobronchiomegaly. Report of five cases and demonstration of familial occurrence. *Amer. Rev. Respir. Dis.* 1965; **91**: 35–50.
[21] Woodring, J.H., Howard II, R.S. and Rehm, S.R. Congenital tracheobronchomegaly (Mounier–Kuhn syndrome): a report of 10 cases and review of the literature. *J. Thorac. Imaging* 1991; **6**: 1–10.
[22] Sane, A.C., Effmann, E.L. and Brown, S.D. Tracheobronchomegaly. The Mounier–Kuhn syndrome in a patient with the Kenny–Caffey syndrome. *Chest* 1992; **102**: 618–619.
[23] Lallemand, D., Chagnon, S. and Buriot, D. *et al.* Trachéomégalie et deficit immunitaire chez l'enfant. *Ann. Radiol.* 1981; **24**: 67–72.
[24] Ratliff, J.L., Campbell, G.D. and Reid, M.V. Tracheobronchomegaly: report of two cases with widely differing symptomatology. *Ann. Otol. Rhinol. Laryngol.* 1977; **86**: 172–175.
[25] Scwartz, M. and Rossoff, L. Tracheobronchomegaly. *Chest* 1994; **106**: 1589–1590.
[26] Schivaram, U., Shivaram, I. and Cash, M. Acquired tracheobronchomegaly resulting in severe respiratory failure. *Chest* 1990; **96**: 491–492.
[27] Vidal, C., Pena, F. and Mosquera, R.M. *et al.* Tracheobronchomegaly associated with interstitial pulmonary fibrosis. *Respiration* 1991; **58**: 2007–2010.
[28] van Klaveren, R.J., Morshuis, W.J. and Lacquet, L.K. *et al.* Congenital bronchial atresia with regional emphysema associated with pectus excavatum. *Thorax* 1992; **47**: 1082–1083.
[29] Malcolm, A.D. Mitral valve prolapse associated with other disorders. Casual coincidence, common link, or fundamental genetic disturbance? *Br. Heart J.* 1985; **53**: 353–362.
[30] Salomon, J., Shah, P.M. and Heinle, R.A. Thoracic skeletal abnormalities in idiopathic mitral valve prolapse. *Am. J. Cardiol.* 1975; **36**: 32–36.
[31] Udoshi, M.B., Shah, A. and Fisher, V.J. *et al.* Incidence of mitral valve prolapse in subjects with thoracic skeletal abnormalities – prospective study. *Am. Heart. J.* 1979; **97**: 303–311.
[32] Margaliot, S.Z., Barzilay, J. and Bar-David, M. Spontaneous pneumothorax and mitral valve prolapse. *Chest* 1986; **89**: 93–94.
[33] Snell, N.J.C. Mitral valve prolapse, spontaneous pneumothorax, and abnormal lung collagen. *Chest* 1987; **91**: 793.

